# Candida pericarditis and tamponade from a malignant pericardio-oesophageal fistula

**DOI:** 10.4102/sajid.v40i1.741

**Published:** 2025-08-31

**Authors:** Nicola K. Wills, Priyadarshini Arnab, Ferdinand Oompie, Nectarios S. Papavarnavas

**Affiliations:** 1 Department of Medicine, Faculty of Health Sciences, University of Cape Town, Cape Town, South Africa; 2 Division of Pulmonology, Department of Medicine, Faculty of Medicine and Health Sciences, Stellenbosch University and Tygerberg Hospital, Cape Town, South Africa; 3 Division of Infectious Diseases and HIV Medicine, Department of Medicine, Faculty of Health Sciences, University of Cape Town, Cape Town, South Africa; 4 Division of Radiology, Faculty of Health Sciences, University of Cape Town, Cape Town, South Africa

**Keywords:** invasive candidiasis, *Candida glabrata*, *Nakaseomyces glabrata*, candida pericarditis, tamponade, malignant oesophago-pericardial fistula

## Abstract

We describe the case of a middle-aged man and long-term smoker, presenting in cardiac tamponade with paroxysmal atrial flutter, with translocation of *Candida glabrata* into the pericardial space as a result of an erosive malignant oesophago-pericardial fistula.

**Contribution:**

The isolation of *Candida* species from the pericardial space is a rare manifestation of invasive candida infection, carrying a high mortality rate, and requires interrogation for possible underlying immune aberrations or mechanical portals of entry.

## Case presentation

A 61-year-old repairman with hypertension and a 30 pack-year cigarette smoking history was admitted to a district hospital in the Western Cape province of South Africa with a 2-week history of chest pain, shortness of breath, cough and night sweats, but no weight loss or tuberculosis contact. His electrocardiogram showed atrial flutter with multi-territory anteroseptal and inferior saddle-shaped ST segment elevation (high-sensitivity troponin testing negative), with an estimated 40% left ventricular ejection fraction on initial echocardiogram, without any significant pericardial or other pathology noted. With a presumed initial diagnosis of heart failure, he received diuretic therapy and was later discharged from hospital for outpatient cardiology review.

**FIGURE 1 (a-c):**
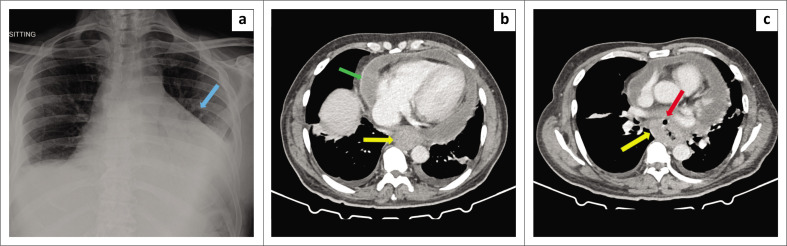
Chest X-ray (a) from day 9 (with clinical and echocardiographic cardiac tamponade), showing traverse, crisp cardiomegaly in keeping with pericardial effusion (blue arrow). Computed tomography (CT) scan of the chest in (b) and (c) showing rim enhancing pericardial effusion (green arrow), circumferencial thickening of the oesophagus (yellow arrows) and an anterior oesophageal air locule suggestive of mural breech into the pericardium (red arrow).

Three days later, he represented to the same facility, with similar complaints now with added concerns of a hospital-acquired pneumonia (Figure 1a). Despite treatment with piperacillin-tazobactam and amikacin, as per protocol in our institution for healthcare-associated infection, the patient decompensated and required inotropic support. The electrocardiogram now showed features of pericarditis with multi-territory saddle-shaped ST elevation and PR depression, with atrial flutter and a variable atrioventricular block. An urgent bedside echocardiogram confirmed a large pericardial effusion. The patient was urgently sent to a tertiary facility where the cardiology team performed pericardiocentesis, draining 800 mL of straw-coloured fluid with immediate resolution of haemodynamic instability and return to sinus rhythm.

Pericardial fluid showed a raised adenosine deaminase (ADA) of 47 U/L, exudative chemistry with a protein of 56 g/L and lactate dehydrogenase of 2240 U/L. A cell count was not available. The patient was empirically treated for *Mycobacterium tuberculosis.* Pericardial fluid culture, however, revealed growth of the fungus *Candida glabrata* (now reclassified as *Nakaseomyces glabrata*), sensitive to micafungin and amphotericin B. In light of the patient’s acute renal injury (peak creatinine 187, GFR 33), he was treated with micafungin for 14 days from positive pericardial culture result. GeneXpert (polymerase chain reaction [PCR]) testing for *M. tuberculosis* on pericardial fluid and sputum was negative – and based on clinical discretion, we discontinued his tuberculosis treatment. Investigation for immunosuppression showed that he was HIV seronegative, with no evidence of endocrine, metabolic or underlying hepato-renal abnormality. He had normochromic normocytic anaemia and a high ferritin 17 997 ug/L (upper limit of normal < 400 ug/L). Bacterial and fungal blood and urine cultures taken prior to initiating antibiotic and antifungal therapy during his second admission were negative.

**FIGURE 2 (a, b):**
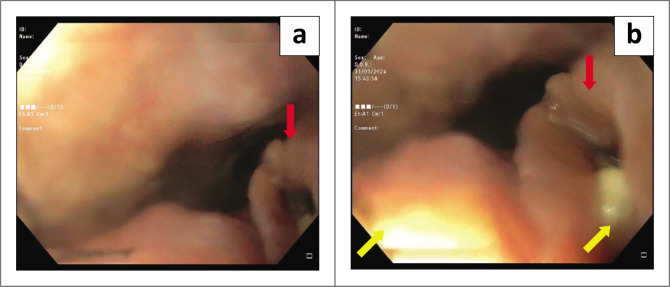
Gastroscopy showing native oesophageal orifice (red arrows) and then defect (yellow arrows) at 30 cm from the incisors. Food stuff and inflammatory exudate can be seen draining from the defect.

A computed tomography (CT) scan (Figure 1b and c) of the chest, abdomen and pelvis revealed a circumferential oesophageal mass with associated necrotic lymph nodes, suggestive of neoplasia. Additionally, a focal anterior mural breech through the tumour to the pericardium and an associated large rim-enhancing pericardial effusion were also noted. Subsequent gastroscopy revealed unilateral external oesophageal compression 18 cm – 35 cm from the incisors (front teeth), and a 5 mm defect, with macroscopically friable and necrotic borders, at 30 cm (Figure 2a and b). A stent was deployed over the defect. Histopathological examination of a biopsy demonstrated a moderately differentiated non-keratinising squamous cell carcinoma with features suspicious for lympho-vascular invasion. Oesophageal biopsies also cultured *C. glabrata* with the same drug susceptibility profile as in the pericardial fluid.

The patient’s inpatient course was complicated by re-accumulation of pericardial effusion and atrial flutter with haemodynamic instability, requiring an urgent left antero-lateral thoracotomy with pericardial drainage and drain placement. Intra-operatively, fibrinopurulent strands were found in the pericardial space with adherence of the oesophagus to the pericardium posteriorly. Pericardial and pleural tissue cultures were sterile.

Further history from family members revealed that he had reported dysphagia for solid foods over the preceding 6 months. The patient was referred to our palliative oncology service, and 2 weeks later, he was managing well with minimal pain and could enjoy most foods without discomfort at home.

## Discussion

Invasive candidiasis describes candida infection, with or without candidaemia, of otherwise normally sterile tissue^1^ and has emerged as an increasing nosocomial concern because of its high associated mortality rate. Risk factors for invasive candidiasis include an intravascular device, recent surgery (particularly abdominal surgery), broad spectrum antibiotics and antifungals, immunosuppressive therapies, malignancies and diabetes mellitus.^2,3^ As the initial echocardiogram did not illustrate a pericardial effusion, we hypothesise that indolent pericardial inflammation was driving our patient’s initial presentation with chest pain and atrial flutter, with subsequent evolution to effusive pericarditis, because of inoculation with *C. glabrata* from the oesophagus through a malignant oesophago-pericardial fistula, and likely hastened by immune aberrations induced by smoking and newly diagnosed metastatic squamous carcinoma.

Candida pericarditis is exceptionally rare; a review published in 2002 identified 30 cases from 1967 onwards.^4^ In this review, the frequency of predisposing conditions or interventions included recent cardiac or thoracic surgery (48%), abdominal surgery (5%), erosive oesophagitis (5%) and immunosuppression with candidaemia (malignancy, steroid therapy, burns, diabetes, alcohol use or autoimmune disease) (39%).^4^ Furthermore, over 60% of patients received broad spectrum antibiotics prior to pericarditis onset, promoting fungal colonisation. Subsequent isolated case reports highlight the rarity of candida pericarditis and underscore the role of underlying immunosuppression or thoraco-abdominal interventions.^5^ While *C. albicans*, the most common candida species, is more frequently identified in candidal pericardial infections,^6,7^ *C. glabrata* is rarely reported.^4^ Emerging data suggest an evolving epidemiological shift with increasing representation of *C. glabrata* and waning dominance of *C. albicans* species in invasive candidiasis.^8^ The comparative virulence, immune-evasive and tissue-invasive capabilities of *C. glabrata*,^9^ as well as its inherent resistance to azole antibiotics, mark this fungus out as a formidable pathogen.

The mortality of candida pericarditis was 70% in the 2002 case series.^4^ The high mortality may relate to delay in diagnosis because of non-specific presentation, as well as cardiac sequelae.^4,6,10^ Cardiac tamponade is a critical consequence of acute fluid collection, raised intra-pericardial pressures and supraventricular tachycardias. Atrial fibrillation and flutter are among the more common electrophysiological consequences of the accumulation of irritant fluid around the left atrium.^11^ During his admission, our patient demonstrated a pattern of recurrent atrial flutter episodes coupled with pericardial inflammation and accumulation or re-accumulation of pericardial fluid, with prompt return to sinus rhythm after pericardiocentesis. Sinus rhythm was then sustained after a pericardial window and closure of the oesophageal defect was performed, and once anti-fungal therapy was established. Morbidity and mortality from invasive candidiasis may be reduced through prompt fungal isolation and drug sensitivity testing, but available laboratory diagnostics have limitations. The sensitivity of blood culture for invasive candidiasis ranges from 21% to 71%, while surrogate markers, such as blood ß-D-glucan lack specificity, and PCR assays are limited by cost and need for validation.^1,2^ The sensitivity of pericardial fluid culture and other blood markers for candida pericarditis is not well described; liver abscess aspirate has a culture positivity rate of approximately 11%.^12^ Clinical suspicion of invasive disease and multi-sample submission for culture is hence essential for prompt diagnosis.^1^ As in this patient, *C. glabrata* did not grow in routine and fungal blood culture despite being cultured from pericardial fluid, followed by oesophageal biopsy, demonstrating the limitations of peripheral blood sampling in the context of deep-seated invasive disease.

Drainage of pericardial collections and prompt, appropriate antifungal therapy are central to optimising treatment outcomes in candida pericarditis.^4^ Optimal duration of therapy is poorly defined,^13^ guided largely by single case reports and series with robust trial data. Most cases relate to *C. albicans* rather than other candida species. International guidelines advise 14 days of appropriate anti-fungal therapy from the first day of persistently negative blood cultures in the case of initial candidaemia,^13,14^ but there is far less evidence to guide treatment duration for organ involvement without candidaemia. Morbidity and mortality rates vary,^5,7,15^ but some case reports indicate good outcomes with a minimum of 4–6 weeks parenteral antifungal therapy followed by oral treatment, when combined with pericardiocentesis or surgical drainage.^4,16^

Prompt recognition of cardiac compromise and emergency therapeutic pericardiocentesis facilitated isolation of *C. glabrata* from pericardial fluid in our patient, enabling early introduction of antifungal therapy prior to diagnostic gastroscopy and palliative oesophageal stenting. We opted for a finite duration of 2 weeks of micafungin therapy, guided by his rapid clinical improvement, definitive drainage of the pericardial collection, subsequent clearance of *C. glabrata* from cultured pericardial samples at surgery, closure of the oesophageal fistula with early source control and his family and our patient’s decision for end-of-life palliation.

## Conclusion

Candida pericarditis, largely caused by *C. albicans*, is rare and usually associated with tissue breaches through recent cardiac, thoracic or abdominal surgery or intervention, or seeding from candidaemia in the context of immunosuppression, often after recent exposure to broad-spectrum antibiotics. In this case, *C. glabrata* was inoculated into the pericardial space causing a life-threatening cardiac tamponade as a consequence of a malignant oesophago-pericardial fistula. Possible reasons for invasive disease must be interrogated if not immediately clear. To promote optimal outcomes, a high clinical index of suspicion, with multi-tissue sampling, is needed for the isolation of invasive candida species and to direct appropriate antifungal and drainage therapy. Current treatment duration for pericardial infection, in the absence of candidaemia, is guided largely by opinion rather than evidence and is an important area for future research.
